# Increased tumor-infiltrating CD45RA^−^CCR7^−^ regulatory T-cell subset with immunosuppressive properties foster gastric cancer progress

**DOI:** 10.1038/cddis.2017.388

**Published:** 2017-08-17

**Authors:** Fang-yuan Mao, Hui Kong, Yong-liang Zhao, Liu-sheng Peng, Weisan Chen, Jin-yu Zhang, Ping Cheng, Ting-ting Wang, Yi-pin Lv, Yong-sheng Teng, Xiao-long Fu, Yu-gang Liu, Xiao-long Wu, Chuan-jie Hao, Nan You, Ping Luo, Pei-wu Yu, Quan-ming Zou, Gang Guo, Yuan Zhuang

**Affiliations:** 1 National Engineering Research Center of Immunological Products, Department of Microbiology and Biochemical Pharmacy, College of Pharmacy, Third Military Medical University, Chongqing, China; 2 Department of General Surgery and Center of Minimal Invasive Gastrointestinal Surgery, Southwest Hospital, Third Military Medical University, Chongqing, China; 3 La Trobe Institute of Molecular Science, School of Molecular Science, La Trobe University, Bundoora, Victoria 3085, Australia; 4 Department of Hepatobiliary Surgery, Xinqiao Hospital, Third Military Medical University, Chongqing, China

## Abstract

Regulatory T cells (Tregs) are major components of tumor-infiltrating immune cells with potent immunosuppressive properties in gastric cancer (GC) microenvironment. However, different subsets of the Tregs and their relevance to GC are unknown. Here, we found that patients with GC showed a significantly higher Tregs infiltration in tumors, and CD45RA^−^CCR7^−^ Treg subset constituted most tumor-infiltrating Tregs. Tumor-infiltrating CD45RA^−^CCR7^−^ Treg subset with an effector/memory phenotype accumulated in tumors and expressed low level of HLA-DR. Gastric tumor-derived TNF-*α* induced CD45RA^−^CCR7^−^ Treg subset with similar phenotype to their status in tumors and inhibited their HLA-DR expression via activating STAT3 phosphorylation. These tumor-associated CD45RA^−^CCR7^−^ Treg subset exerted superior immunosuppressive properties to effectively suppress CD8^+^ T cells’ anti-tumor function including CD8^+^ T-cell IFN-*γ* and granzyme B (GrB) production as well as CD8^+^ T-cell proliferation *in vitro*, and also contributed to the growth and progression of human gastric tumors *in vivo*, via IL-10 secretion and cell–cell contact mechanisms. Moreover, increased tumor-infiltrating CD45RA^−^CCR7^−^ Treg subset as well as higher intratumoral CD45RA^−^CCR7^−^ Treg/CD8^+^ T-cell ratio was associated with advanced disease progression and reduced GC patient survival. This study therefore identifies a novel immunosuppressive pathway involving CD45RA^−^CCR7^−^ Treg subset development within the GC microenvironment. Efforts to inhibit this pathway may therefore prove a valuable strategy to prevent, and to treat this immune suppressive of GC.

Gastric cancer (GC) is the fourth most common cancer worldwide, and is also a very aggressive disease with poor prognosis,^1^ which has great relevance with the GC immunosuppressive microenvironment that promotes tumor progression and attenuates therapeutic efficacy in GC patients.^2^

T cells are normally a major component of tumor-infiltrating immune cells.^3^ Recently, a subset of regulatory T cells (Tregs) with potent immunosuppressive properties has been detected in human GC, and increased such Tregs was also associated with poor prognosis of GC patients.^4^ Although most data on circulating^5^ or tumor-infiltrating^6^ Tregs belonged to CD4^+^CD25^+^Foxp3^+^ T cells, little is known about the phenotype, regulation, function, and clinical relevance of Treg subsets in human gastric tumors.

Generally, Tregs, like other T cells, can be divided into CD45RA^+^ naïve and CD45RA^−^ memory subtypes.^7^ The memory population can be further characterized into: CD45RA^−^CCR7^+^ (central memory), CD45RA^−^CCR7^−^ (effector/memory), CD45RA^+^CCR7^−^ (terminally differentiated).^8^ CD45RA is located on naïve T cells and when they are activated or transformed into memory cells, CD45RA is lost. CCR7, a seven transmembrane G protein-coupled chemokine receptor, is essential for the migration of T cells and dendritic cells (DCs) to the surrounding lymphoid tissue or the site of immune response by binding to its ligands,^9^ mainly expressed on the cell surface of T cells^10^ and DCs,^11^ as well as some tumor cells. However, the regulatory mechanisms of such Treg subsets, especially in human GC, need to be further elucidated.

In this study, we investigated the number, distribution and phenotype of Tregs and their subsets in GC. We show that a novel Treg subset with CD45RA^−^CCR7^−^ effector/memory phenotype constitutes most tumor-infiltrating Tregs and that this Treg subset is highly enriched in GC with disease progression and negatively correlated with patient survival following surgery. Moreover, we demonstrated that gastric tumor-derived TNF-*α* efficiently induced CD45RA^−^CCR7^−^ Treg subset and inhibited HLA-DR expression on these cells by inducing signal transducer and activator of transcription 3 (STAT3) phosphorylation. In turn, this CD45RA^−^CCR7^−^ Treg subset suppresses CD8^+^ T-cell anti-tumor function via IL-10 secretion and cell–cell contact mechanisms, and, in doing so, contribute to the immunosuppression and GC progression.

## Results

### Tregs are enriched in GC with a classical profile

**Figure 1:**
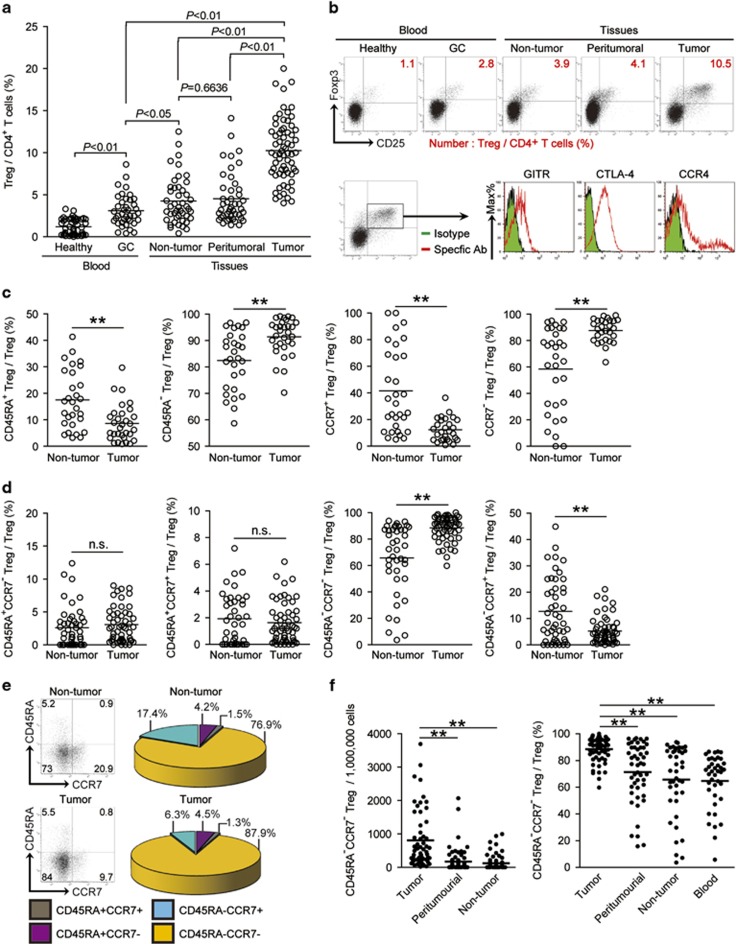
CD45RA^−^CCR7^−^ effector/memory Treg subset constituted the majority of Tregs and accumulated in GC. (**a**) Treg percentage in CD4^+^ T cells in each tissue of patients with GC by gating on CD3^+^CD4^+^CD25^+^Foxp3^+^ cells. Cumulative results from 51 GC patients and 45 healthy donors are shown. (**b**) Dot plots of surface and intracellular molecule staining for Tregs gating on CD4^+^ T cells, and multicolor flow cytometry for markers or subpopulations of intratumoral Tregs. The horizontal bars and each ring in panel b represent mean values and one patient. GITR, glucocorticoid-induced tumor necrosis factor receptor; CTLA-4, cytotoxic T lymphocyte-associated antigen-4. (**c**) Statistics analysis of CD45RA^+^ and CD45RA^−^ Treg percentage or CCR7^+^ and CCR7^−^ Treg percentage in total Tregs in tumor and non-tumor tissues of GC patients. (**d**) Statistics analysis of the percentages of CD45RA^+^CCR7^+^, CD45RA^−^CCR7^+^, CD45RA^−^CCR7^−^, and CD45RA^+^CCR7^−^ Treg subsets in total Tregs in non-tumor or tumor tissues. (**e**) Dot plots of surface staining and pie charts summarizing for CD45RA^+^CCR7^+^, CD45RA^−^CCR7^+^, CD45RA^−^CCR7^−^, and CD45RA^+^CCR7^−^ Treg subsets by gating on total Tregs. (**f**) The number of CD45RA^−^CCR7^−^ Treg subset per million total cells, or CD45RA^−^CCR7^−^ Treg subset percentage in total Tregs in blood or each tissue of patients with GC by counting or gating on Tregs. The horizontal bars and each ring or dot in panels **a**, **c**, **d** and **f** represent mean values and one patient. *, *P*<0.05; **, *P*<0.01, and n.s., *P*>0.05 for groups connected by horizontal lines

To evaluate the potential role of Tregs and its subsets in human GC, we first gated CD4^+^CD25^+^Foxp3^+^ T lymphocytes as Tregs and analyzed the Treg percentage within the total CD4^+^ T-cell populations from peripheral blood, non-tumor, peritumoral, and tumor tissues of GC patients. Peripheral blood from healthy donors was included as a control. Notably, patients with GC showed a higher frequency of Tregs in peripheral blood than healthy donors (Figures 1a and b). Within the patient cohort, tumors contained a significantly higher proportion of Tregs than non-tumor, or peritumoral tissues (Figures 1a and b), suggesting a potential role for Tregs in the GC microenvironment. We also performed immuno-phenotyping of intratumoral Tregs to better understand their likely status. Gating on intratumoral Tregs, we found that Tregs expressed glucocorticoid-induced tumor necrosis factor receptor-related protein (GITR), CTLA-4, and CCR4 (Figure 1b), indicating that most intratumoral Tregs were classical immunosuppressive lymphocytes. On the basis of our observation, we conclude that tumor-infiltrating Tregs accumulated in the GC microenvironment and may perform immunosuppressive functions in GC patients.

### CD45RA^−^CCR7^−^ effector/memory phenotype Treg subset constitutes most tumor-infiltrating Tregs and accumulated in GC

To study phenotypic features of Tregs at tumor site, we gated on intratumoral Tregs, and found that most Foxp3^+^ Tregs belonged to the CD45RA^−^ memory T-cell phenotype (Figure 1c). Next, we also found that intratumoral Tregs expressed little homing molecule CCR7 (Figure 1c), suggesting that they may be permanent residents, and have high potential to exert effector function in GC. Finally, we examined Treg subsets according to the expression of both CD45RA and CCR7, and found that most intratumoral Tregs displayed a CD45RA^−^CCR7^−^ effector/memory phenotype (Figures 1d and e), whereas non-tumor tissue-derived Tregs displayed largely more CD45RA^−^CCR7^+^ central memory phenotype (Figures 1d and e). In addition, there were no differences of CD45RA^+^CCR7^−^ and CD45RA^+^CCR7^+^ Treg subset percentages between tumors and non-tumor tissues (Figures 1d and e), which constituted no more than 10% total Tregs. Within the patient cohort, tumors contained a significantly higher CD45RA^−^CCR7^−^ Treg subset percentage than peritumoral, non-tumor tissues and peripheral blood (Figure 1f and Supplementary Figure 1). Similar observations were made when analyzing the number of CD45RA^−^CCR7^−^ Treg subset per million total cells in gastric tissues (Figure 1f). Taken together, our data indicate that CD45RA^−^CCR7^−^ effector/memory Treg subset accounted for most Tregs accumulated in the GC microenvironment.

### CD45RA^−^CCR7^−^ Treg subset in GC is induced by tumor-derived TNF-*α*

**Figure 2:**
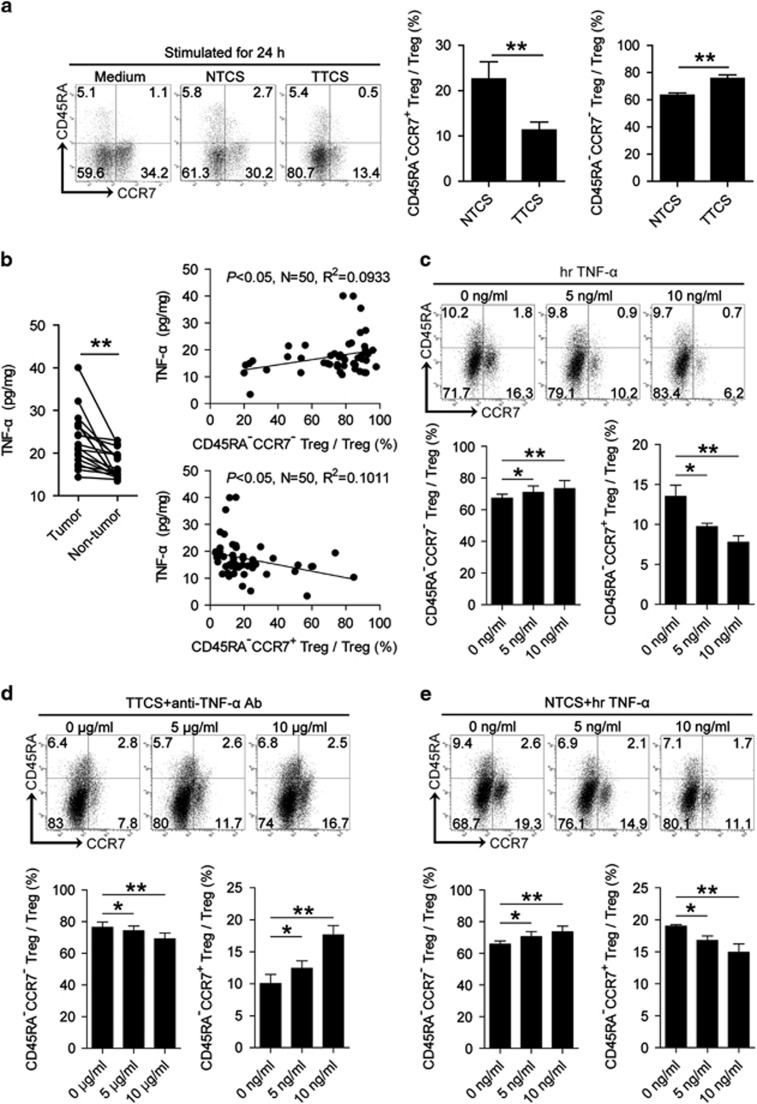
Tumor-derived TNF-*α* induces CD45RA^−^CCR7^−^ Treg subset. (**a**) Dot plots and statistics analysis of CD45RA^−^CCR7^+^ and CD45RA^−^CCR7^−^ Treg subsets after Tregs exposed to autologous TTCS and NTCS for 24 h. (**b**) TNF-*α* concentration between autologous tumor and non-tumor tissues (*n*=18) was analyzed. The correlations between TNF-*α* and CD45RA^−^CCR7^−^ or CD45RA^−^CCR7^+^ Treg subsets in GC were analyzed (*n*=50). (**c–e**) Dot plots and statistics analysis of CD45RA^−^CCR7^+^ and CD45RA^−^CCR7^−^ Treg subsets after Tregs exposed to TNF-*α* (**c**), or after Tregs exposed to TTCS with anti-TNF-*α* antibody (**d**), or after Tregs exposed to NTCS with TNF-*α* (**e**) for 24 h. Each dot in panel **b** represents one patient. *, *P*<0.05; **, *P*<0.01, and n.s., *P*>0.05 for groups connected by horizontal lines

Given the superior ability of Tregs to exhibit CD45RA^−^CCR7^−^ effector/memory phenotype in GC, we hypothesized that tumor microenvironment itself might play important roles in this process. To test this hypothesis, peripheral derived-Tregs were purified and cultured with tumor tissue culture supernatants (TTCS) or non-tumor tissue culture supernatants (NTCS) for 24 h. Interestingly, more TTCS-conditioned Tregs showed CD45RA^−^CCR7^−^ phenotype than NTCS-conditioned Tregs (Figure 2a), suggesting that tumors released soluble factors which induced CD45RA^−^CCR7^−^ Treg subset pool. As CD45RA^−^CCR7^−^ and CD45RA^−^CCR7^+^ Treg subsets contribute about 90% Tregs (Figure 2), and CCR7 expression on immune cells is regulated by pro-inflammatory cytokine TNF-*α*,^12^ we hypothesized that TNF-*α* might regulate CCR7 expression on Treg subsets in GC. Firstly, we found a significantly increased TNF-*α* production (Figure 2b) as well as a positive correlation between CD45RA^−^CCR7^−^ Treg subset and TNF-*α* within gastric tumors (Figure 2b); next, to evaluate the potential role of TNF-*α* in CD45RA^−^CCR7^−^ Treg subset induction, we co-cultured TNF-*α* and purified-Tregs, and found that TNF-*α* significantly increased the frequency of CD45RA^−^CCR7^−^ Treg subset whereas inhibited CD45RA^−^CCR7^+^ Treg subset (Figure 2c). To further evaluate tumor-derived TNF-*α* in this induction, we added neutralizing antibody against TNF-*α* into our TTCS and purified-Treg co-culture system. Interestingly, antibody blockade of TNF-*α* efficiently decreased the frequency of CD45RA^−^CCR7^−^ Treg subset (Figure 2d). Consistent with these findings, provision of exogenous TNF-*α* significantly promoted the generation of CD45RA^−^CCR7^−^ Treg subset in the NTCS and purified-Treg co-culture system (Figure 2e). Taken together, our data demonstrated that gastric tumor-derived TNF-*α* plays an essential role in the induction of CD45RA^−^CCR7^−^ Treg subset *in vitro*, and suggest that a similar process might operate *in vivo*.

### Tumor-derived TNF-*α* activates STAT3 phosphorylation to induce CD45RA^−^CCR7^−^ Treg subset in GC

**Figure 3:**
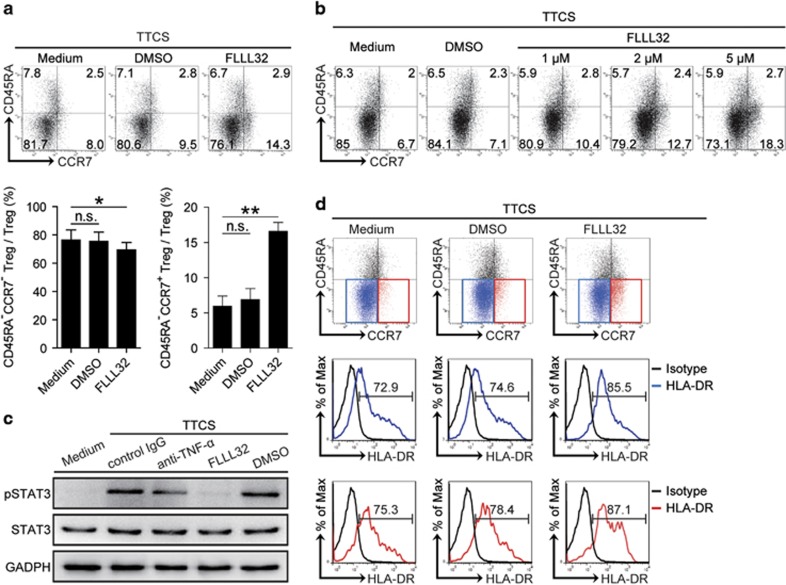
Tumor-derived TNF-*α* induces CD45RA^−^CCR7^−^ Treg subset via STAT3 phosphorylation. (**a**,**b**) Dot plots and statistics analysis of CD45RA^−^CCR7^+^ and CD45RA^−^CCR7^−^ Treg subset after Tregs exposed to TTCS for 24 h with or without pre-treated with different concentrations of STAT3 phosphorylation inhibitor FLLL32 for 1 h. (**c**) STAT3 and p-STAT3 proteins in Tregs exposed to autologous TTCS, or TTCS with anti-TNF-*α* antibody or TTCS pre-treated with FLLL32 (an STAT3 inhibitor) or DMSO were analyzed by western blot. (**d**) Dot plots and HLA-DR expression of CD45RA^−^CCR7^+^ and CD45RA^−^CCR7^−^ Treg subset after Tregs exposed to TTCS for 24 h with or without pre-treated with STAT3 phosphorylation inhibitor FLLL32 for 1 h. *, *P*<0.05; **, *P*<0.01, and n.s., *P*>0.05 for groups connected by horizontal lines

The activation of inflammation-associated signaling pathways is implicated in the regulation of T-cell functions. To see which signaling pathways might operate in the CD45RA^−^CCR7^−^ Treg subset induction, we first pre-treated normal Tregs with corresponding inhibitors including FLLL32 (an STAT3 inhibitor), BAY 11-7082 (an I*κ*B*α* inhibitor), SP600125 (a JNK inhibitor), or SB203580 (a MAPK inhibitor) and so on, and then exposed them to TTCS. The results showed that only abolishing the phosphorylation of STAT3 with inhibitor FLLL32 effectively suppressed CD45RA^−^CCR7^−^ Treg subset induction (Figure 3a and Supplementary Figure 2). Moreover, this CD45RA^−^CCR7^−^ Treg subset induction by STAT3 phosphorylation had a dose-dependent effect (Figure 3b). Furthermore, STAT3, a direct TNF-*α* downstream substrate, was predominantly phosphorylated in Tregs after treatment with TTCS, and this phosphorylation was abolished when blocking TNF-*α* or abolishing STAT3 phosphorylation with inhibitor FLLL32 (Figure 3c). Moreover, blocking STAT3 phosphorylation could effectively increase HLA-DR expression on CD45RA^−^CCR7^−^ Treg subsets induced by TTCS (Figure 3d), indicating that tumor-induced intracellular STAT3 phosphorylation inhibited HLA-DR expression on CD45RA^−^CCR7^−^ Treg subset. These data imply that STAT3 phosphorylation is crucial for the induction of CD45RA^−^CCR7^−^ Treg subset by TNF-*α* in GC.

### CD45RA^−^CCR7^−^ Treg subset exhibits higher immunosuppressive effects on CD8^+^ T cells via IL-10 and cell-contact mechanisms

**Figure 4:**
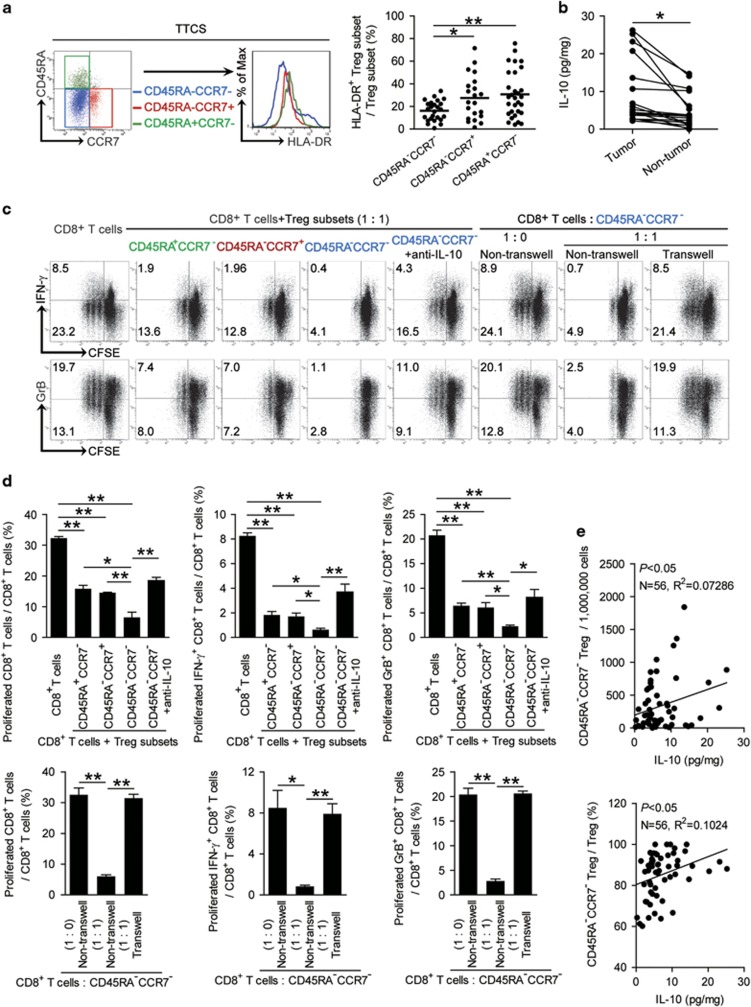
CD45RA^−^CCR7^−^ Treg subset suppress CD8^+^ T-cell functions via IL-10 secretion and cell–cell contact mechanisms. (**a**) Representative surface staining and statistics analysis of the expression of HLA-DR on CD45RA^−^CCR7^−^ (blue), CD45RA^−^CCR7^+^ (red) or CD45RA^+^CCR7^−^ (green) Treg subsets. (**b**) IL-10 concentration between autologous tumor and non-tumor tissues (*n*=19) was analyzed. (**c** and **d**) CD8+ T cells and Treg subsets were co-cultured and assessed by transwell assay as described in Materials and Methods. Representative data (**c**) and statistical analysis (**d**) of CD8+ T-cell proliferation and IFN-*γ*, GrB production were shown (*n*=3). (**e**) The correlations between IL-10 and CD45RA^−^CCR7^−^ Treg subset in GC were analyzed. Results are expressed as percentage of CD45RA^−^CCR7^−^ Treg subset in total Tregs or the number of CD45RA^−^CCR7^−^ Treg subset per million total cells and IL-10 concentration in gastric tumor tissues. The horizontal bars and each dot in panel **a** represent mean values and one patient. *, *P*<0.05; **, *P*<0.01, and n.s., *P*>0.05 for groups connected by horizontal lines

To see functional difference of Treg subsets above in GC, we first found that a weaker HLA-DR expression (Figure 4a) as well as CD80 and CD86 expression (Supplementary Figure 3) on CD45RA^−^CCR7^−^ Treg subsets when compared with CD45RA^+^CCR7^−^ or CD45RA^−^CCR7^+^ Treg populations. Next, we purified peripheral autologous CD8^+^ T cells and co-cultured with TTCS-conditioned CD45RA^−^CCR7^−^, CD45RA^−^CCR7^+^ or CD45RA^+^CCR7^−^ Treg subsets respectively for 5 days. Interestingly, CD45RA^−^CCR7^−^ Treg subset was more suppressive on CD8^+^ T cell IFN-*γ* and granzyme B (GrB) production as well as their proliferation than CD45RA^+^CCR7^−^ or CD45RA^−^CCR7^+^ Treg subsets (Figures 4c and d).

As for the significantly higher IL-10 production detected in tumor tissues than that in non-tumor tissues (Figure 4b) and a positive correlation between CD45RA^−^CCR7^−^ Treg infiltration and IL-10 production within gastric tumors observed (Figure 4e). Next, to see whether IL-10 plays role in this suppression of CD45RA^−^CCR7^−^ Treg subset on CD8^+^ T cells’ immunity, we added neutralizing antibodies against IL-10 in CD8^+^ T cell/CD45RA^−^CCR7^−^ Treg subset co-culture. Interestingly, antibody blockade of IL-10 efficiently attenuated such CD8^+^ T-cell suppression mediated by CD45RA^−^CCR7^−^ Treg subset (Figures 4c and d). To further explore whether other mechanism may also contribute to this suppression, we performed transwell assays and found that cell–cell contact was also involved in this suppression of CD8^+^ T-cell proliferation, IFN-*γ* and GrB production (Figures 4c and d and Supplementary Figure 4). Taken together, our data demonstrate that CD45RA^−^CCR7^−^ Treg subset exhibits high immunosuppressive effects on CD8^+^ T cells via IL-10 secretion and cell–cell contact mechanisms.

### Blockade of immunosuppressive CD45RA^−^CCR7^−^ Treg subset inhibits tumor growth and GC progression

**Figure 5:**
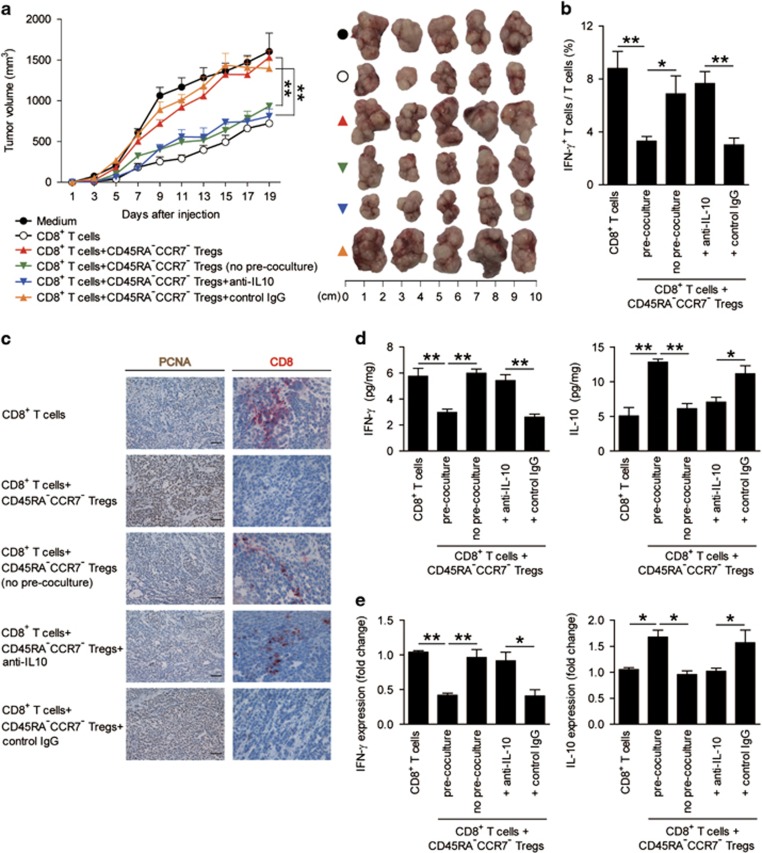
Blockade of immunosuppressive CD45RA^−^CCR7^−^ Tregs inhibits tumor growth and GC progression *in vivo*. (**a**) Mice were injected with human SGC7901 cells, as described in Materials and methods. The control animals (●) received no further injections. The experimental treatments entailed injections with CD8^+^ T cells alone (○) or in combination with TTCS-conditioned CD45RA^−^CCR7^−^ Tregs with (▴) or without (▾) pre-coculturing, or TTCS-conditioned CD45RA^−^CCR7^−^ Tregs pre-treated with an anti-IL-10 antibody (▾) or a control IgG (▴). The illustrated data represent tumor volumes (five mice in each group). The day of T-cell injection was counted as day 0. The tumors were excised and photographed 19 day after injecting T cells. (**b**–**e**) IFN-*γ*-producing T-cell response (**b**) in spleens, proliferating cell nuclear antigen (PCNA) (brown) expression or CD8^+^ T-cell infiltration (red) (**c**), and production (**d**) or expression (**e**) of IFN-*γ* and IL-10 in tumors of mice on day 19 after T-cell injection were compared. Scale bars: 100 *μ*m. *P<0.05; **P<0.01, and n.s., *P*>0.05 for groups connected by horizontal lines

To test the suppressive effect and underlying mechanisms of CD45RA^−^CCR7^−^ Treg subset on CD8^+^ T cells *in vivo*, we first generated TTCS-conditioned CD45RA^−^CCR7^−^ Tregs as described above, and pre-cocultured these CD45RA^−^CCR7^−^ Tregs with or without polyclonal CD8^+^ T cells, or cocultured CD45RA^−^CCR7^−^ Tregs with polyclonal CD8^+^ T cells with IL-10 blocking antibody or control IgG for 24 h, and then injected them together into our established human non-obese diabetic/severe combined immunodeficient (NOD/SCID) mice bearing SGC-7901-derived GC. As expected, mice without CD8^+^ T-cells transfusions, mice treated with CD8^+^ T cells plus CD45RA^−^CCR7^−^ Tregs or control IgG-treated CD45RA^−^CCR7^−^ Tregs showed tumor growth and disease progression (Figure 5a). Consistent with a vital role in assisting tumors of CD45RA^−^CCR7^−^ Tregs *in vivo*, mice treated with no pre-cocultured CD8^+^ T cells/CD45RA^−^CCR7^−^ Tregs, or with CD8^+^ T cells plus IL-10 blocking antibody-treated CD45RA^−^CCR7^−^ Tregs showed reduced tumor volumes and disease progression at each measurement time point from day 9 (Figure 5a). Moreover, mice treated with no pre-cocultured CD8^+^ T cells/CD45RA^−^CCR7^−^ Tregs, or with CD8^+^ T cells plus IL-10 blocking antibody-treated CD45RA^−^CCR7^−^ Tregs, also showed an increased IFN-*γ*^+^ T-cell response in spleens (Figure 5b), as well as an increased CD8^+^ T-cell infiltration (Figure 5c) and IFN-*γ* expression/production (Figures 5d and e) in tumors, and a decreased tumor cell proliferation (Figure 5c) and TNF-*α* expression/production in tumors (Supplementary Figure 5), compared with the mice treated with CD8^+^ T cells plus CD45RA^−^CCR7^−^ Tregs or control IgG-treated CD45RA^−^CCR7^−^ Tregs. These findings suggest that tumor-induced CD45RA^−^CCR7^−^ Treg subset suppresses CD8^+^ T-cell immunity *in vivo* dependent on IL-10 secretion and cell–cell contact mechanisms and thereby contribute to tumor growth and GC progression.

### Increased tumor-infiltrating CD45RA^−^CCR7^−^ Treg subset correlates with tumor stage and GC patient poor survival

**Figure 6:**
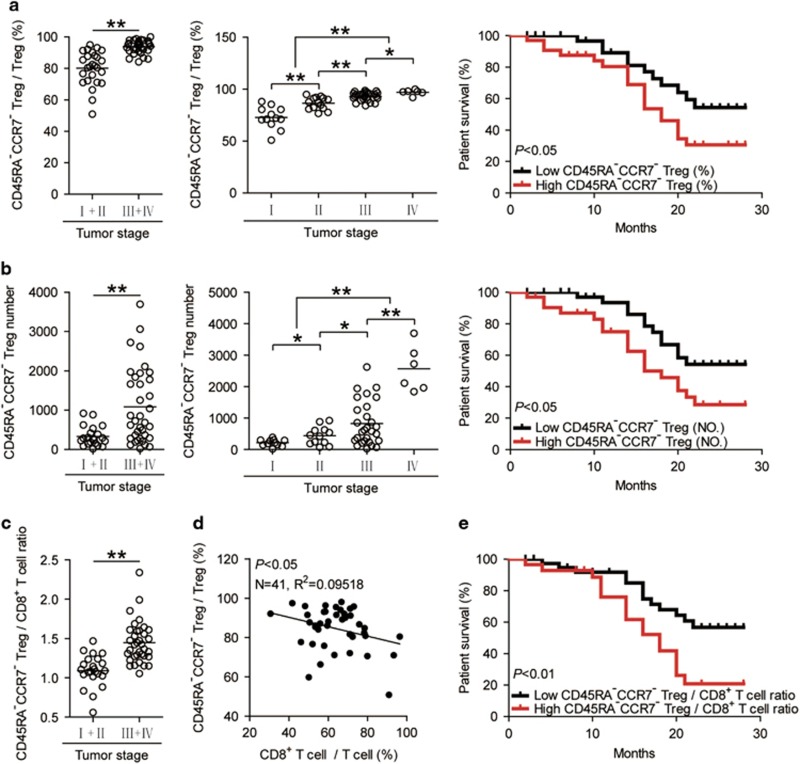
Increased tumor-infiltrating CD45RA^−^CCR7^−^ Treg subset correlates with tumor stage and patient poor survival. (**a**,**b**) Percentages of CD45RA^−^CCR7^−^ Treg subset in Tregs (**a**) and the number of CD45RA^−^CCR7^−^ Treg subset per million total cells (**b**) among TNM stage were compared. Kaplan–Meier plots for patient survival by median CD45RA^−^CCR7^−^ Treg subset percentage (%, 91.3%) (**a**) or median CD45RA^−^CCR7^−^ Treg subset number (NO., 457.94 per million) (**b**). Survival significantly decreased as a function of CD45RA^−^CCR7^−^ Treg subset percentage⩾91.3% or CD45RA^−^CCR7^−^ Treg subset number⩾457.94. (**c**) CD45RA^−^CCR7^−^ Treg/CD8^+^ T-cell ratio among TNM stage were compared. (**d**) The correlations between CD45RA^−^CCR7^−^ Tregs and CD8^+^ T cells in gastric tumors were analyzed. Results are expressed as the ratio of the percentages of CD8^+^ T cells in T cells and the percentages of CD45RA^−^CCR7^−^ Tregs in Tregs in gastric tumors. (**e**) Kaplan–Meier plots for patient survival by median CD45RA^−^CCR7^−^ Treg/CD8^+^ T-cell ratio (1.289). Survival significantly decreased as a function of CD45RA^−^CCR7^−^ Treg/CD8^+^ T-cell ratio ⩾1.289. The horizontal bars and each ring in panels **a**–**c** represent mean values and one patient. *, *P*<0.05; **, *P*<0.01, and n.s., *P*>0.05 for groups connected by horizontal lines

Finally, we studied whether increased CD45RA^−^CCR7^−^ Treg subset percentage were associated with tumor stage (Figure 6a), suggesting that CD45RA^−^CCR7^−^ Treg subset accumulates at tumor site during tumor progression. We also evaluated the prognostic value of intratumoral CD45RA^−^CCR7^−^ Treg subset percentage on the survival of GC patients. Comparing patients with high (⩾91.3% median level) *versus* low (<91.3%) CD45RA^−^CCR7^−^ Treg subset percentage level, the 30-month survival rate was significantly higher for those within the high CD45RA^−^CCR7^−^ Treg subset percentage group (Figure 6a). Similar observations were made when analyzing the number of CD45RA^−^CCR7^−^ Treg subset per million total cells in gastric tumor tissues (Figure 6b). In keeping with this finding, an increased CD45RA^−^CCR7^−^ Treg subset percentage/number was correlated with increased tumor size and advanced lymphatic invasion (Supplementary Figure 6).

As for an inverse correlation between intratumoral CD45RA^−^CCR7^−^ Treg subset and CD8^+^ T cells (Figure 6d), next, we evaluated the prognostic value of intratumoral CD45RA^−^CCR7^−^ Treg/CD8^+^ T-cell ratio on the survival of GC patients. Comparing patients with high *versus* low levels of CD45RA^−^CCR7^−^ Treg/CD8^+^ T-cell ratio, the 30-month survival rate was significantly lower for those with higher CD45RA^−^CCR7^−^ Treg/CD8^+^ T-cell ratio groups (Figure 6e). Taken together, these findings suggest that increased intratumoral CD45RA^−^CCR7^−^ Treg subset is associated with tumor progression and poor survival for GC patients.

## Discussion

Many studies focused on tumor-mediated immunosuppression and the subsequent tumor progression over the past decades.^13^ Tregs were major component of tumor-infiltrating immune cells with potent immunosuppressive properties in tumor microenvironment.^14^ In this study, we have shown that within GC a novel subset of CD45RA^−^CCR7^−^ Tregs play immunosuppressive roles on promoting tumor progression. Although Tregs have already been described in patients with GC tumors,^15^ and a higher frequency of Treg cells with effector phenotypes have recently been described in late stage GC,^16^ to our knowledge this is the first demonstration of a statistically significant correlation between prevalent CD45RA^−^CCR7^−^ Treg subset infiltration with effector/memory phenotype in gastric tumors and their poor prognosis; it is also the first demonstration for tumor-derived TNF-*α* to induce CD45RA^−^CCR7^−^ Treg subset which then suppress CD8^+^ T cells’ anti-tumor function connecting mechanistically the pathological role of CD45RA^−^CCR7^−^ Treg subset within the tumor microenvironment.

Tregs are MHC class II restricted CD4^+^ T cells with the ability to suppress the effector functions of immune cells.^17^ Nevertheless, subsets of Tregs that play key roles in tumor immune escape remain largely undefined. In this study, we firstly observed a CD45RA^−^ memory-phenotyping tumor-infiltrating Tregs in GC. In ovarian tumors, memory Tregs comprise two populations that represent the counterparts of conventional CCR7^+^ central memory and CCR7^−^ effector/memory subsets.^18^ Therefore, we determine whether such a mechanism existed and was also responsible for the CD45RA^−^ memory Tregs inside our gastric tumors. We further re-examined tumor-infiltrating Tregs according to both CD45RA and CCR7, and confirmed most Tregs showing a CD45RA^−^CCR7^−^ effector/memory phenotype, which is in accordance with the results on Tregs in squamous cell head/neck carcinoma regarded as ‘effector’ phenotype.^19^ Collectively, these expression profiles reveal that CD45RA^−^CCR7^−^ tumor-infiltrating Treg subset exhibits a phenotype for effector functional features.

Tumor-related inflammation and immunosuppression have been proposed as the hallmarks of cancer.^20^ Recently, in human GC, a subset of CD127^low/−^ Tregs could exert immunosuppressive properties, although the detailed regulatory mechanisms were not elucidated.^21^ In our case, we subsequently identified a novel regulatory mechanism for tumor-derived pro-inflammatory TNF-*α*: it promotes CD45RA^−^CCR7^−^ Treg subset induction and downregulates HLA-DR expression by activating intracellular STAT3. STAT3 is critical component of downstream substrates of inflammatory cytokine TNF-*α*,^22^ and is implicated in the regulation of T-cell functions in tumor immunology.^23^ Here, we identify STAT3 as a key factor within CD45RA^−^CCR7^−^ Treg subset induction. It has also been reported that the pro-inflammatory environment may induce the increase of CD45RA^−^CCR7^−^ Tregs in synovial fluid from patients with rheumatoid arthritis,^24^ which resembles our data on CD45RA^−^CCR7^−^ Treg subset induction by GC tumor-derived pro-inflammatory elements. Together with other reported observations that in rheumatoid arthritis CD127^low^ Tregs accumulated in inflammatory environments and exerted an inhibitory effect on effector T-cell proliferation,^25^ it is highly likely that CD45RA^−^CCR7^−^ Treg subset represents a shared effect of immune suppression in human GC.

Tregs and its subsets are considered as mixed populations, which may mediate immunosuppression through co-stimulation or post-translational modification of the TCR.^26^ Most studies have shown that the immunosuppressive activities of Tregs require direct cell–cell contact, which suggests that they function either through cell-surface receptors and/or through the release of soluble mediators.^27^ Therefore we have shown that tumor-derived TNF-*α* effectively inhibit HLA-DR expression on CD45RA^−^CCR7^−^ Treg subset; moreover, functional experiments showed that tumor-associated CD45RA^−^CCR7^−^ Treg subset reduced the detectable cytolytic molecules granzyme B and IFN-*γ* in CD8^+^ T cells and inhibited their proliferation via both cell–cell contact and IL-10 secretion. Most studies have shown that the immunosuppressive activities of Tregs require direct cell–cell contact, cell-surface receptors and/or through the release of short-lived soluble mediators.^28^ Notably, our transwell assays revealed the same mechanism. Together with other reported observations on Treg cell subsets such as CD127^low^Tregs accumulated synovial fluid from patients with rheumatoid arthritis and exerted an inhibitory effect on effector T-cell proliferation,^25^ it is highly likely that CD45RA^−^CCR7^−^ Treg cell subsets represent a shared mechanism of immune suppression in human GC.

Most importantly, our findings also shed light on the clinical relevance of CD45RA^−^CCR7^−^ Treg subsets in GC. CD45RA^−^CCR7^−^ Treg cell subsets are increased in GC tumors and correlated with advanced tumor progression and poorer patient survival. Since the clinical outcome for GC patients remains poor and that few prognostic factors currently exist for this disease following surgery,^18^ these Treg subsets might be used as clinical markers for GC patients in the future.

In summary, based on our *in vitro* and *in vivo* data, we identified in human GC an immunosuppressive pathway, which leads to CD45RA^−^CCR7^−^ Treg subset induction by tumor-derived TNF-*α* and subsequent CD8^+^ T cells’ suppression and correlates with tumor progression and poor survival. Thus, immune-boosting therapeutic strategies aimed at interfering with this negative pathway may prove beneficial in GC patients.

## Materials and methods

### Patients and tissue samples

Fresh peripheral blood, tumor (homogeneous cellularity, without foci of necrosis, etc.), peritumoral, or autologous non-tumor gastric tissues (peritumoral tissues, 3–4 cm distant from tumor site; non-tumor tissues, at least 5 cm distant from tumor site) were obtained from patients who underwent surgical resection at the Southwest Hospital of Third Military Medical University. None of the patients had received radiotherapy or chemotherapy before surgery. Individuals with autoimmune diseases, infectious diseases, and multiple primary cancers were excluded. Peripheral blood from 45 healthy volunteers was used as controls. The clinical stages of tumors were determined according to the TNM classification system of International Union Against Cancer (Edition 7). Characteristics of the study subjects are summarized in Supplementary Table 1. The study was approved by the Ethics Committee of the Southwest Hospital of Third Military Medical University, and written informed consent was obtained from all individuals.

### Antibodies and other reagents

The antibodies for flow cytometry: for human, anti-CD3-APC-H7 and anti-IFN-*γ*-PE-Cy7 from BD Pharmingen (San Jose, CA, USA); anti-CD4-FITC, anti-CD25-PE, anti-Foxp3-APC, anti-CD45RA-PE-Cy7, anti-HLA-DR-PerCP-Cy5.5, anti-CCR7-PerCP-Cy5.5, anti-GITR-PE-Cy7, and CD8-PerCP-Cy5.5 from eBioscience (CA, USA); anti-Foxp3-Alexa Fluor 488, anti-CTLA-4-PE-Cy7, anti-CCR4-PE-Cy7, anti-CD44-FITC, anti-CD69-APC-Cy7, and anti-granzyme B-APC from Biolegend (San Diego, CA, USA). The antibodies for immunohistochemical staining were as follows: anti-human proliferating cell nuclear antigen (PCNA) from Santa Cruz (Santa Cruz, CA, USA); anti-human CD8 from Dako(Glostrup, Denmark). Horseradish peroxidase (HRP)-conjugated secondary antibodies were from Zhongshan Biotechnology (Beijing, China). The antibodies for neutralizing and blocking were as follows: anti-human TNF-*α* from R&D Systems; anti-human IL-10 from R&D Systems (Minneapolis, MN, USA). The Abs for western blot: anti-human STAT3 from Cell signaling technology (Beverly, MA, USA); anti-human p-STAT3 (Y705) from Cell signaling technology; HRP-conjugated secondary antibodies were from Zhongshan Biotechnology. Purified anti-CD3 and anti-CD28 Abs were from Biolegend. ELISA kits for human TNF-*α* and human IL-10 were from eBioscience, ELISA kits for human IFN-*γ* were from R&D Systems. Collagenase IV, DNase I, DMSO, phorbol 12-myristate 13-acetate (PMA), and ionomycin were from Sigma-Aldrich (St. Louis, MO, USA). Golgistop was from BD Pharmingen. EnVision G2 System/AP Rabbit/Mouse (Permanent Red) was from Dako. The potent STAT3 inhibitor FLLL32 was from MedKoo Biosciences (Morrisville, NC, USA). I*κ*B*α* inhibitor BAY 11-7082, JNK inhibitor SP600125, and MAPK inhibitor SB203580 were from Calbiochem (Temecula, CA, USA). Carboxyfluorescein succinimidyl ester (CFSE) was from eBioscience. TRIzol reagent was from Invitrogen (Grand Island, NY, USA). Protein Extraction Reagent was from Pierce (Waltham, MA, USA). SuperSignal West Dura Extended Duration Substrate kit was from Thermo (Waltham, MA, USA). Ficoll-Paque Plus was from GE Healthcare (Pittsburg, PA, USA). All recombinant cytokines and chemokines were from PeproTech (Rocky Hill, USA).

### Isolation of single cells from GC tissues

In brief, fresh tumor, peritumoral, and non-tumor tissues were washed three times in RPMI 1640 before cut into small pieces. The specimens were then collected in RPMI 1640 containing 1 mg/ml collagenase IV and 10 mg/ml DNase I and mechanically dissociated by using the gentle MACS Dissociator (Miltenyi Biotec, Bergisch Gladbach, GER). Dissociated cell suspensions were further incubated 1 h at 37 °C under continuous rotation and filtered through 70-*μ*m cell strainers to obtain cell suspensions.

### Tissue culture and preparation of TTCS and NTCS

TTCS or NTCS were prepared by plating autologous tumor or non-tumor gastric tissues in 1 ml RPMI 1640 supplemented with 10% fetal calf serum (FCS) (Gibco, Waltham, MA, USA) for 48 h. Then, the supernatant was centrifuged and collected.

### *In vitro* generation of Tregs

Peripheral blood mononuclear cells (PBMCs) from healthy donors were isolated by Ficoll density gradient centrifugation. CD4^+^ T cells were sorted from PBMCs by negative selection with the EasySep human CD4^+^ T-cell enrichment kit (Stem Cell Technologies). CD4^+^CD25^−^CD44^−^CD69^−^ naïve T cells were further separated from CD4^+^ T cells by (FACSAria II) (BD Biosciences). Purified naïve CD4^+^ T cells were re-suspended at 2 × 10^5^ cells/well in RPMI 1640 containing 10% FCS and seeded in 96-well round bottom plates supplemented with human recombinant (hr) IL-2 (20 IU/ml) and hr TGF-*β*1 (100 ng/ml), in the presence of anti-CD3 (2 *μ*g/ml) and anti-CD28 antibodies (1 *μ*g/ml). On day 5, the cells were analyzed for surface CD25 and intracellular Foxp3 expression by flow cytometry. The sorted cells in all experiments were used unless their viability was determined >95% and their purity was determined >97%.

### Treg stimulation

Normal Tregs were FACS-sorted (FACSAria II) (BD Biosciences) as CD4^+^CD25^+^ generated T cells from blood of healthy donors as described above, and were stimulated with 50% TTCS or 50% autologous NTCS for 24 h, or were stimulated with 50% TTCS with a neutralizing antibody against human TNF-*α* (5, 10 *μ*g/ml), or 50% autologous NTCS with hr TNF-*α* (5, 10 ng/ml) for 24 h. After stimulation, the cells were collected for flow cytometric analysis and western blot. For the signaling pathway inhibition experiments, these cells were pre-treated with 5 *μ*l FLLL32 (an STAT3 inhibitor), BAY 11-7082 (an I*κ*B*α* inhibitor), SP600125 (a JNK inhibitor), or SB203580 (a MAPK inhibitor) (the final concentration of all the inhibitors was 20 *μ*M) for 1 h, then were stimulated with 50% TTCS or hr TNF-*α* (10 ng/ml) for 24 h and collected as above. Since the inhibitors were dissolved in DMSO, parallel cell groups were treated with DMSO (5 *μ*l) or culture media as controls.

### Treg subset-CD8^+^ T-cell co-culture

10^5^ bead-purified peripheral CD8^+^ T cells from healthy donors (Stem Cell Technologies, Vancouver, British Columbia, Canada) were labeled with CFSE and cultured in RPMI 1640 containing 10% FCS containing hr IL-2 (20 IU/ml), anti-CD3 (2 *μ*g/ml) and anti-CD28 (1 *μ*g/ml) antibodies. Normal Tregs were FACS-sorted (FACSAria II) (BD Biosciences) as CD4^+^CD25^+^ generated T cells from autologous blood of healthy donors as described above, and were stimulated with 50% TTCS for 24 h. Then, TTCS-conditioned CD45RA^−^CCR7^−^ CD45RA^+^CCR7^−^ or CD45RA^−^CCR7^+^ Treg subsets were further FACS-sorted and added into CD8^+^ T cells at 1 : 1 ratio in the presence or absence of neutralizing antibody against IL-10 (10 *μ*g/ml). In transwell experiments, CFSE-labeled CD8^+^ T cells were seeded in the lower chamber coated with anti-CD3 (2 *μ*g/ml) and anti-CD28 (1 *μ*g/ml) antibodies; TTCS-conditioned CD45RA^−^CCR7^−^ Treg subset was then added either in the lower or upper chamber. After 5-day incubation, the supernatants were collected for ELISA, and the cells were analyzed by flow cytometry for CFSE dilution and intracellular cytokine staining.

### *In vivo* tumor inhibition assay

All animal experiments were approved by the Animal Ethical and Experimental Committee of Third Military Medical University. 10^6^ GC cells (SGC-7901) in 100 *μ*l of buffered saline were subcutaneously injected into the axillary tissues of female NOD/SCID mice (5–7 week, one tumor per mouse). Peripheral CD8^+^ T cells from healthy donors were bead-purified (Stem Cell Technologies, Vancouver, British Columbia, Canada), and TTCS-conditioned CD45RA^−^CCR7^−^ Treg subset was generated from autologous blood and FACS-sorted as described above. Then, 1 × 10^6^ polyclonal-stimulated (2 *μ*g/ml anti-CD3 and 1 *μ*g/ml anti-CD28) CD8^+^ T cells were co-cultured with or without TTCS-conditioned CD45RA^−^CCR7^−^ Treg subset at a 1:1 ratio in the presence or absence of a neutralizing antibody against human IL-10 (10 *μ*g/ml) or a control IgG (10 *μ*g/ml) for 24 h, and were subsequently injected into the peritoneum in 100 *μ*l of buffered saline on day 10 after tumor cell inoculation. Then, mice were injected intraperitoneally with 20 *μ*g of neutralizing antibody against human IL-10 or a control IgG every 2 days until the mice were killed. Tumor size was measured every 2 days by two independent observers using callipers fitted with a vernier scale. Tumor volume was calculated based on three perpendicular measurements. Once the mice were killed, tumors were photographed, and were further paraformaldehyde-fixed for immunohistochemical staining and cut for RNA and protein extraction, and spleens were dissociated into single cells for flow cytometric analysis.

### Flow cytometric analysis

Flow cytometric analysis was performed according to standard protocols. For intracellular molecule measurements, the cells were stimulated for 5 h with PMA (50 ng/ml) plus ionomycin (1 *μ*g/ml) in the presence of Golgistop. Intracellular cytokine staining was performed after fixation and permeabilization, using Perm/Wash solution (BD Pharmingen). Cells were analyzed by flow cytometry with FACSCanto II (BD Biosciences). Data were analyzed with Flowjo software (TreeStar) or FACSDiva software (BD Biosciences).

### RNA extraction and real-time PCR

RNA of biopsy specimens were extracted with TRIzol reagent. The RNA samples were reversed transcribed to cDNA with PrimeScript RT reagent Kit (TaKaRa, Dalian, China). Real-time PCR was performed on the IQ5 (Bio-Rad, Hercules, CA, USA) with the Real-time PCR Master Mix (Toyobo, Osaka, Japan) according to the manufacturer’s specifications. Expression of TNF-*α*, IL-10, and IFN-*γ* was measured using the SYBR green method with primers (Supplementary Table 2). Human GAPDH served as the normalizers. The relative gene expression was expressed as fold change calculated by the ΔΔCt method.

### ELISA

Human gastric tissues from specimens and mouse tumor tissues were collected, homogenized in 1 ml sterile Protein Extraction Reagent, and centrifuged. Tissue supernatants were collected for ELISA. Concentrations of TNF-*α*, IL-10 and IFN-*γ* and in the tissue supernatants and concentrations of IFN-*γ* (R&D Systems, Minneapolis, MN, USA) in the T-cell culture system supernatants were determined using ELISA kits according to the manufacturer’s instructions.

### Western blot analysis

Western blot assays were performed on 10–15% SDS-PAGE gels using equivalent amounts of cell lysate proteins of samples. Five percent skimmed milk or 3% BSA was used for blocking the PDF membranes. Human STAT3 and p-STAT3 were detected with anti-STAT3 and anti-p-STAT3 antibodies respectively. This was followed by incubation with HRP-conjugated secondary antibodies. Bound proteins were visualized by using SuperSignal West Dura Extended Duration Substrate kit.

### Immunohistochemistry

Paraformaldehyde-fixed and paraffin-embedded samples were cut into 5 *μ*m sections. The sections were incubated with mouse anti-human PCNA or anti-human CD8 antibodies, and then were stained by HRP anti-mouse IgG or using EnVision G2 System/AP Rabbit/Mouse (Permanent Red) followed by diaminobenzidine. All the sections were finally counterstained with haematoxylin and examined using a microscope (Nikon Eclipse 80i; Nikon, Tokyo, Japan).

### Statistical analysis

All results are summarized as mean±S.E.M., and statistical analysis was performed with the SPSS statistical software (version 13.0). Student *t*-test was generally used to analyze the differences between two groups, but when the variances differed, the Mann–Whitney U test was used. Correlations between parameters were assessed using the Pearson correlation analysis and linear regression analysis as appropriate. Overall patient survival was defined as the interval between date of surgery and date of death or last follow-up, which ever occurred earlier. Cumulative survival time was calculated by the Kaplan–Meier method, and survival was measured in months; the log-rank test was applied to compare between two groups. All data were analyzed using two-tailed tests, and *P*<0.05 was considered statistically significant.

## Publisher’s Note

Springer Nature remains neutral with regard to jurisdictional claims in published maps and institutional affiliations.
