# Supplementary material for: Increased tumor-infiltrating CD45RA−CCR7− regulatory T-cell subset with immunosuppressive properties foster gastric cancer progress
Source: Cell Death Dis. 2017 Aug 17;8(8):e3002–. doi: 10.1038/cddis.2017.388 (PMC5596574; doi:10.1038/cddis.2017.388)
Supplement: Supplementary Table 1 [file cddis2017388x1.doc]

**Supplementary Table 1.** Clinical characteristics of 72 patients with gastric cancer

| Variables | No. of patients |
| --- | --- |
| Gender (male/female) | 51/21 |
| Age (years; median, range) | 57, 32-78 |
| *H.pylori* Ab (negative/positive) | 17/55 |
| CEA (U/L; <5/≥5) | 54/18 |
| Tumor size (cm; <5/≥5) | 54/18 |
| Lymphatic invasion (absent/present) | 18/54 |
| Vascular invasion (absent/present) | 67/5 |
| Tumor (T) invasion (T1+T2/T3+T4) | 33/39 |
| Lymphoid Nodal (N) status (N0+N1/N2+N3) | 45/27 |
| Distant metastasis (M) status (M0/M1) | 67/5 |
| TNM stage (Ⅰ+Ⅱ/Ⅲ+Ⅳ) | 32/40 |
| CD45RA^-^CCR7^-^ Treg cell subset percentage^a^ (median, range) | 91.3, 50.9-100 |
| CD45RA^-^CCR7^-^ Treg cell subset number^b^ (median, range) | 457.94, 18.12-3962.67 |

^a^CD45RA^-^CCR7^-^ Treg cell subset percentage was acquired on CD45RA^-^CCR7^-^ Tregs in total Tregs that gated on CD4^+^CD25^+^Foxp3^+^ Tregs of tumor tissues. ^b^CD45RA^-^CCR7^-^ Treg cell subset number was counted as the number of CD45RA^-^CCR7^-^ Tregs per million total cells of tumor tissues. CEA, carcinoembryonic antigen; *H.pylori* Ab, *Helicobacter pylori* antibody.
