# Supplementary material for: Increased tumor-infiltrating CD45RA−CCR7− regulatory T-cell subset with immunosuppressive properties foster gastric cancer progress
Source: Cell Death Dis. 2017 Aug 17;8(8):e3002–. doi: 10.1038/cddis.2017.388 (PMC5596574; doi:10.1038/cddis.2017.388)
Supplement: Supplementary Figure Legends [file cddis2017388x2.doc]

Figure S1. CD45RA^-^CCR7^-^ Treg subset with effector/memory phenotype exhibits the most Tregs and accumulated in GC. (A) Dot plots of surface staining for CD45RA and/or CCR7 expression on Tregs in tumor tissues. (B) CD45RA^-^CCR7^-^ Treg subset percentage in lymphocytes in each tissue of patients with GC. The horizontal bars and each ring or dot in panels B represent mean values and 1 patient. **, *P*<0.01 for groups connected by horizontal lines.

Figure S2. Tumor-derived TNF-α induces CD45RA^-^CCR7^-^ Treg subset via STAT3 phosphorylation. Dot plots of CD45RA^-^CCR7^+^ and CD45RA^-^CCR7^-^ Treg subsets after Tregs exposed to TTCS for 24 h with or without pre-treated with BAY 11-7082 (an IκBα inhibitor), SP600125 (a JNK inhibitor) SB203580 (a MAPK inhibitor), or FLLL32 (an STAT3 inhibitor) (the final concentration of all the inhibitors was 20 μM) for 1 h.

Figure S3. Representative surface staining and statistics analysis of the expression of CD80 and CD86 on CD45RA^-^CCR7^-^ (blue), CD45RA^-^CCR7^+^ (red) or CD45RA^+^CCR7^-^ (green) Treg subsets.

Figure S4. CD45RA^-^CCR7^-^ Treg subset suppresses on CD8^+^ T cells’ immunity via IL-10 secretion and cell-contact mechanisms. CD8^+^ T cells and Treg subsets were co-cultured (A) and assessed **by transwell assay** (B) **as described in** Materials and Methods. Representative data and statistical analysis of IFN-γ production in T-cell culture were shown (n=3). *, *P*<0.05, **, *P*<0.01 for groups connected by horizontal lines.

Figure S5. Blockade of immunosuppressive CD45RA^-^CCR7^-^ Tregs inhibits tumor growth and GC progression *in vivo*. Mice were injected with human SGC-7901 cells, as described in Materials and methods. The control animals received no further injections. The experimental treatments entailed injections with CD8^+^ T cells alone or in combination with TTCS-conditioned CD45RA^-^CCR7^-^ Tregs with or without pre-coculturing, or TTCS-conditioned CD45RA^-^CCR7^-^ Tregs pre-treated with an anti-IL-10 antibody or a control IgG. The illustrated data represent tumor volumes (5 mice in each group). The day of T cell injection was counted as day 0. The tumors were excised and photographed 19 day after injecting T cells. TNF-α production (A) or expression (B) in tumors of mice on day 19 after T cell injection were compared. *, *P*<0.05, **, *P*<0.01 for groups connected by horizontal lines.

Figure S6. CD45RA^-^CCR7^-^ Treg subset percentage (A) and number (B) and their potential correlations with clinical parameters. *, *P*<0.05; **, *P*<0.01, and n.s, *P*>0.05 for groups connected by horizontal lines. Each dot represents one patient. CEA, carcinoembryonic antigen; *H.pylori* Ab, *Helicobacter pylori* antibody.
