# Supplementary material for: Increased tumor-infiltrating CD45RA−CCR7− regulatory T-cell subset with immunosuppressive properties foster gastric cancer progress
Source: Cell Death Dis. 2017 Aug 17;8(8):e3002–. doi: 10.1038/cddis.2017.388 (PMC5596574; doi:10.1038/cddis.2017.388)
Supplement: Supplementary Table 2 [file cddis2017388x3.doc]

**Supplementary Table 2. Primer and probe sequences for real-time PCR analysis**

| Gene | Primer or probe | Sequence 5′→3′ |
| --- | --- | --- |
| *H. pylori* 16S rDNA | forward | TTTGTTAGAGAAGATAATGACGGTATCTAAC |
|  | reverse | CATAGGATTTCACACCTGACTGACTATC |
|  | probe | CGTGCCAGCAGCCGCGGT |
| *H. pylori* *cagA* | forward | GAGTCATAATGGCATAGAACCTGAA |
|  | reverse | TTGTGCAAGAAATTCCATGAAA |
| Human β-actin | forward | TTCCTTCCTGGGCATGGAGTCC |
|  | reverse | TGGCGTACAGGTCTTTGCGG |
| Human IL-22 | forward | GACAAGTCCAACTTCCAG |
|  | reverse | GCTCACTCATACTGACTC |
| Human IL-22R1 | forward | AGCCCTGCAAGGCAGAAATG |
|  | reverse | GATTGTGAAACTGGCCAGGAATG |
| Human CXCL2 | forward | CCCAAGTTAGTTCAATCCTG |
|  | reverse | TTCCTCAGCCTCTATCACAG |
| Mouse β2-microglobulin | forward | CCTGCAGAGTTAAGCATGCCAG |
|  | reverse | TGCTTGATCACATGTCTCGATCC |
|  | probe | TGGCCGAGCCCAAGACCGTCTAC |
| Mouse Sry | forward | TGGGACTGGTGACAATTGTC |
|  | reverse | GAGTACAGGTGTGCAGCTCT |
| Mouse IL-22 | forward | ATACATCGTCAACCGCACCTTT |
|  | reverse | AGCCGGACATCTGTGTTGTTAT |
| Mouse IL-22R1 | forward | CTACGTGTGCCGAGTGAAGA |
|  | reverse | AAGCGTAGGGGTTGAAAGGT |
| Mouse S100A8 | forward | GGAAATCACCATGCCCTCTAC |
|  | reverse | GCCACACCCACTTTTATCACC |
| Mouse S100A9 | forward | AACATCTGTGACTCTTTAGCCTTG |
| Human TNFα  Human IL-10  Human IFN gamma  Human GAPDH | reverse  forward  reverse  forward  reverse  forward  reverse  forward  reverse | ACTGTGCTTCCACCATTTGTCT  CGGGATCCGAAATTGACACAAGTGGACC  CGGAATTCCTCCCAAATAAATACATTCATCTG  GACTTTAAGGGTTACCTGGGTTGC  ATTCTTCACCTGCTCCACGGC  AGCTCTGCATCGTTTTGGGTT  GTTCCATTATCCGCTACATCTGAA  ACCCAGAAGACTGTGGATGG  CAGTGAGCTTCCCGTTCAG |

For the probes, a FAM fluorescent reporter is coupled to the 5' end, and a TAMRA quencher is coupled to the 3' end.
