# Supplementary figures and images for: Increased tumor-infiltrating CD45RA−CCR7− regulatory T-cell subset with immunosuppressive properties foster gastric cancer progress
Source: Cell Death Dis. 2017 Aug 17;8(8):e3002–. doi: 10.1038/cddis.2017.388 (PMC5596574; doi:10.1038/cddis.2017.388)

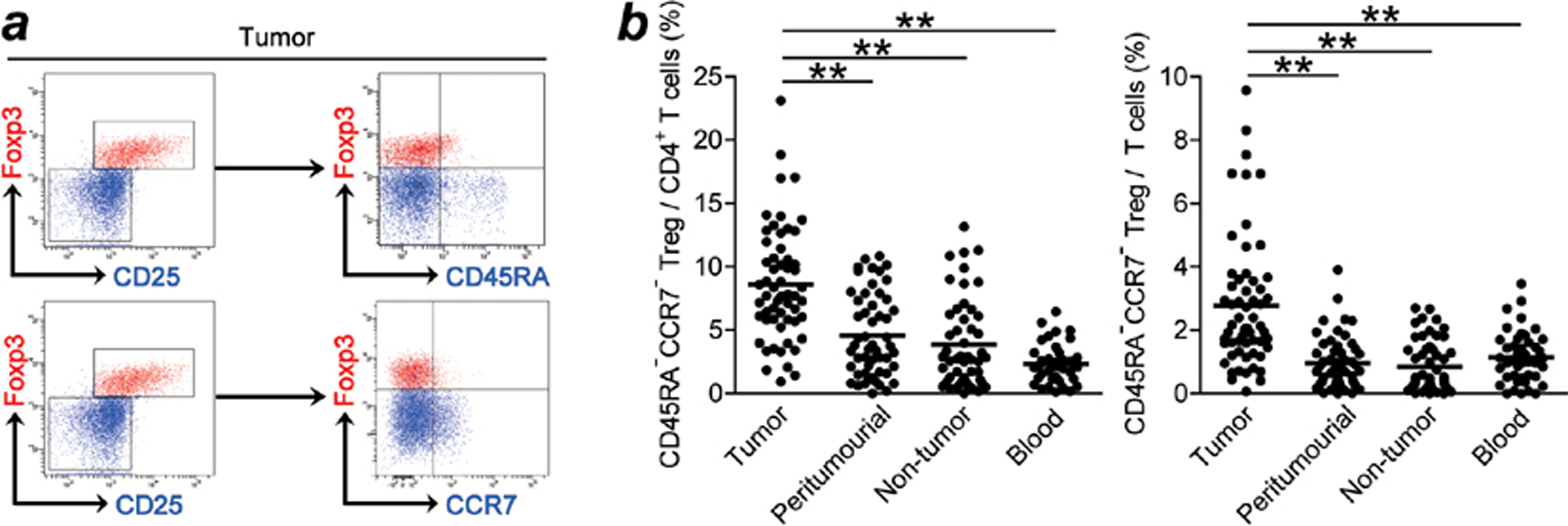

Supplement: Supplementary Figure 1 [file cddis2017388x4.tif]

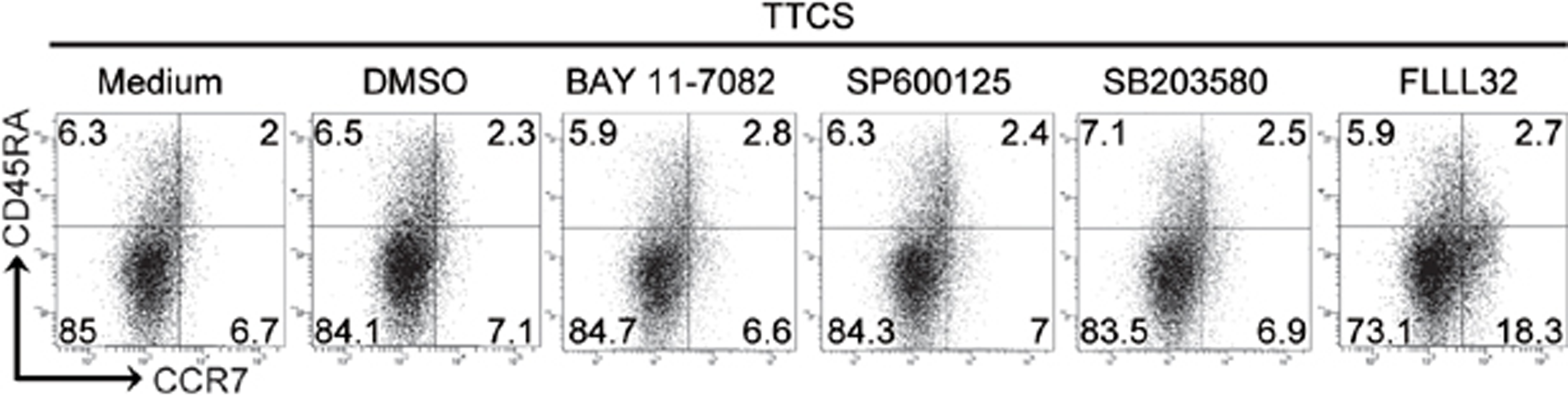

Supplement: Supplementary Figure 2 [file cddis2017388x5.tif]

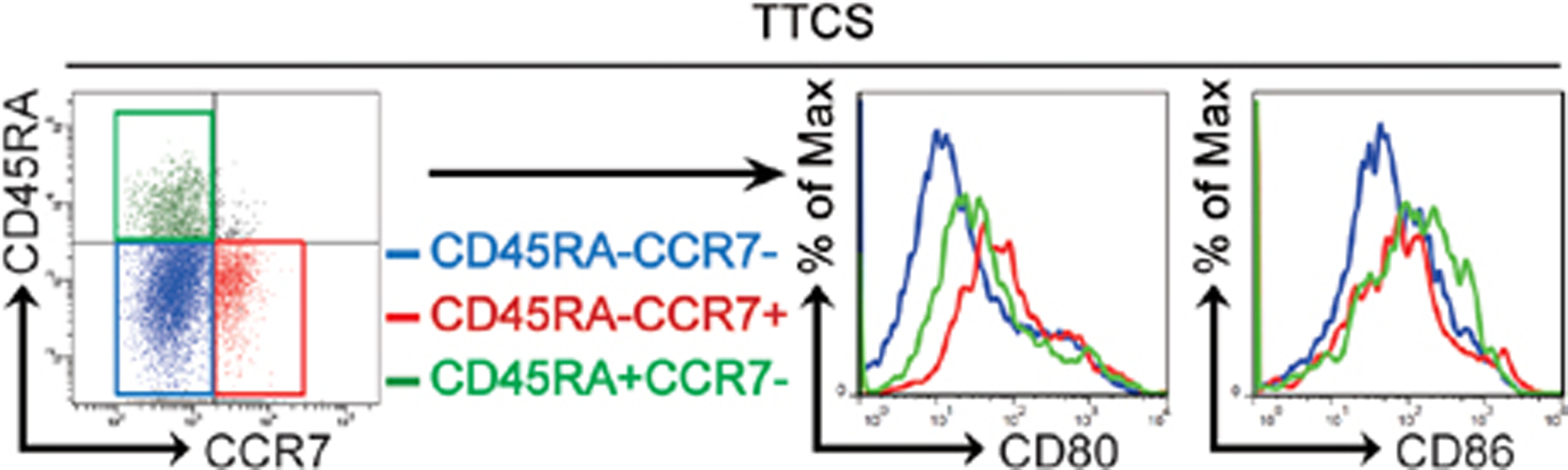

Supplement: Supplementary Figure 3 [file cddis2017388x6.tif]

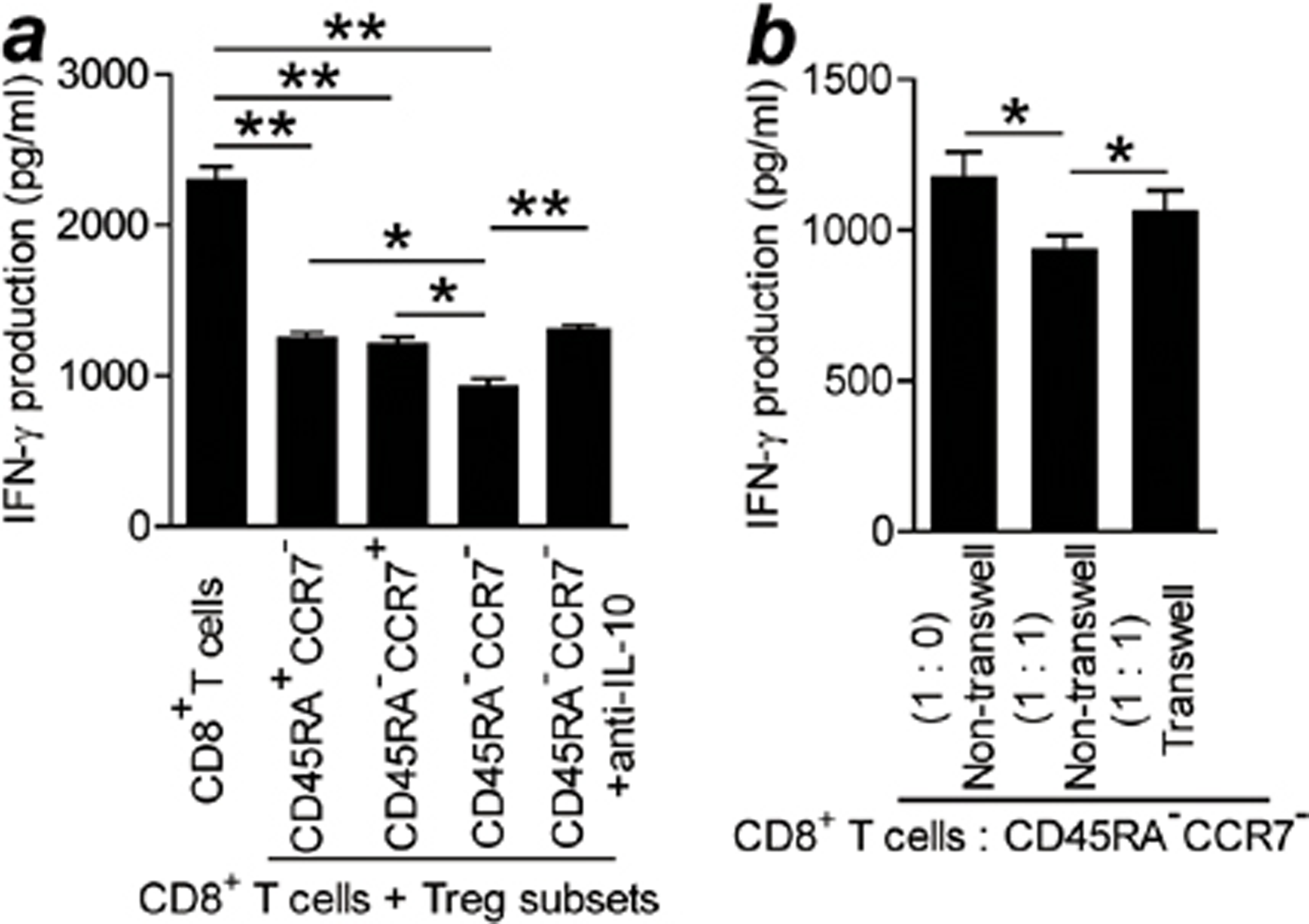

Supplement: Supplementary Figure 4 [file cddis2017388x7.tif]

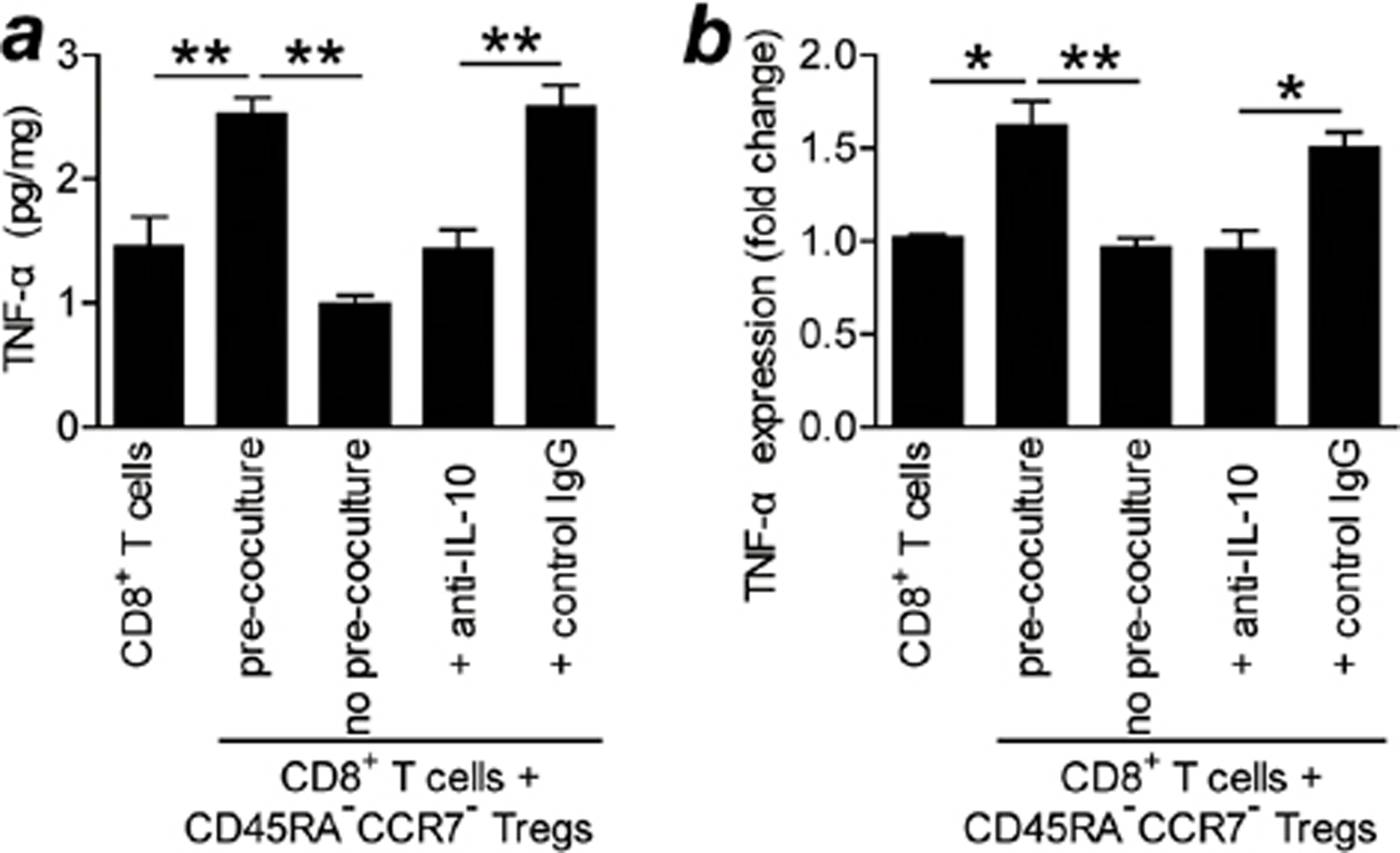

Supplement: Supplementary Figure 5 [file cddis2017388x8.tif]

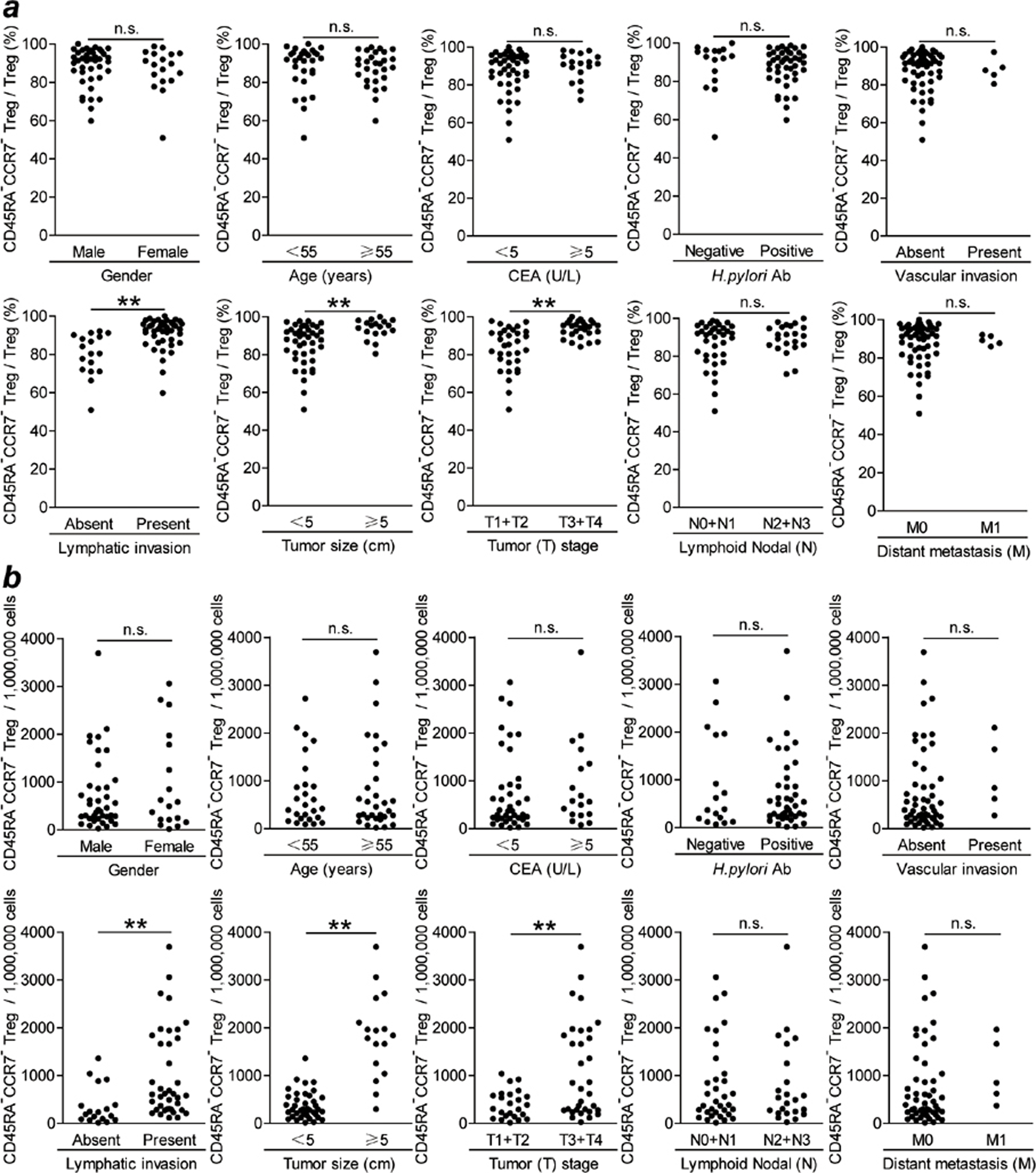

Supplement: Supplementary Figure 6 [file cddis2017388x9.tif]
